# Supplementary material for: Characterization of the Glutamine Synthetase Gene Family in Wheat (Triticum aestivum L.) and Expression Analysis in Response to Various Abiotic Stresses
Source: Int J Mol Sci. 2025 Sep 26;26(19):9403. doi: 10.3390/ijms26199403 (PMC12524364; doi:10.3390/ijms26199403)
Supplement: Supplementary file 1 [file ijms-26-09403-s001.zip › ijms-3805086-supplementary figures caption.pdf]

Figure S1. Phylogenetic analysis of GS from *T.aestivum*, *Z.mays*, *O.sativa*, *A.thaliana*, *M.truncatula*, and *P.vulgaris*. A phylogenetic tree was constructed using MEGA 12.0 with 1000 bootstrap replicates. Distinct colors denote different groups or subgroups within a family.

Figure S2. Putative motifs of the TaGS protiens.

Figure S3. Comparative analysis of domains in TaGS protiens.

Figure S4. Schematic representation of the inter-chromosomal relationships of TaGS genes in *T.aestivum*.

Figure S5. Analysis of cis-acting elements in the promoter region of TaGS. Different color blocks represent various cis-acting elements, respectively.

Figure S6. Prediction of transcription factor regulatory network analysis of TaGS genes based on an Arabidopsis association model.

Figure S7. Predicted post-translational modification sites for TaGS protein phosphorylation, glycosylation, S-nitrosylation, palmitoylation, ubiquitination, and sumoylation.

Figure S8. The histochemical GUS staining of transgenic plants Arabidopsis harboring *proTaGS1-2-4D::GUS* leaves under various biotic stress conditions.

These leaves of forty-day-old transgenic Arabidopsis harboring *proTaGS1-2-4D::GUS* were collected and transferred to control (½ MS liquid medium, 22°C), salt stress (½ MS liquid medium containing 200 mM sodium chloride, 22°C), heat stress (½ MS liquid medium, 40°C), drought stress (½ MS liquid medium containing 20% m/V PEG 6000, 22°C), and cold stress (½ MS liquid medium, 4°C) treatment for 6 h. These samples were incubated in GUS solution for overnight at 37°C. The leaves were destained using 70% ethanol, and then photographed using a stereomicroscope. The leaves and leaf vascular tissue were treated with control (a and f), salt stress (b and g), heat stress (c and h), drought stress (d and i) and cold stress (e and j), respectively. Scale bars = 2 mm in (a-e) and 1 mm in (f-j).

Figure S9. Gene Ontology enrichment analyses for the TaGS-interacting proteins.
