# Supplementary figures and images for: Characterization of the Glutamine Synthetase Gene Family in Wheat (Triticum aestivum L.) and Expression Analysis in Response to Various Abiotic Stresses
Source: Int J Mol Sci. 2025 Sep 26;26(19):9403. doi: 10.3390/ijms26199403 (PMC12524364; doi:10.3390/ijms26199403)

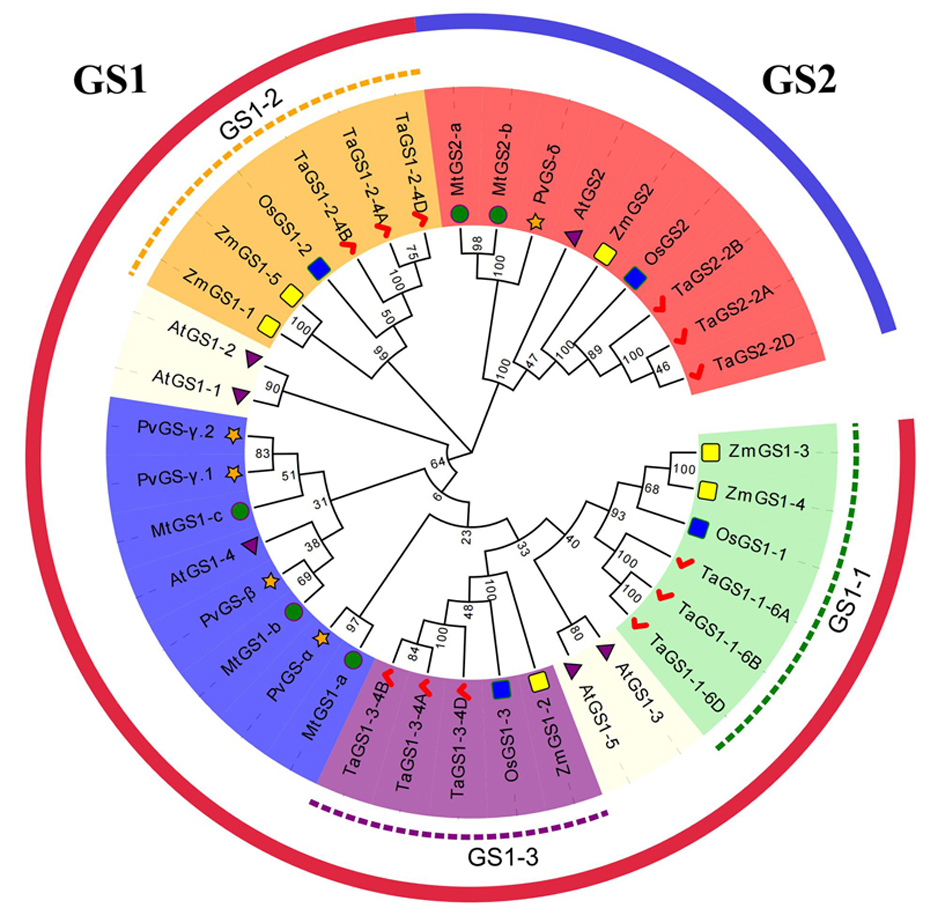

Supplement: Supplementary file 1 [file ijms-26-09403-s001.zip › Supplementary Figure S1.jpg]

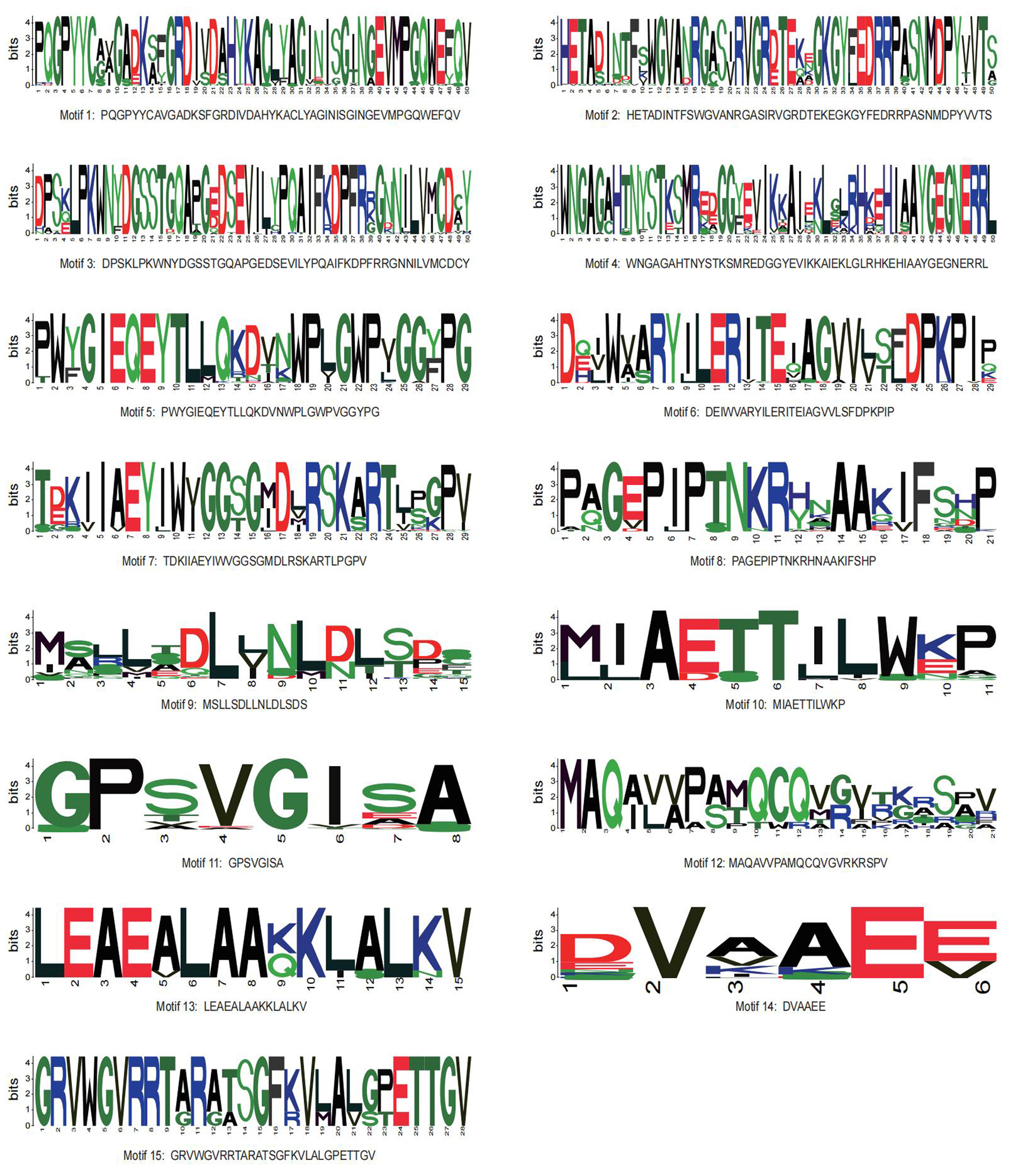

Supplement: Supplementary file 1 [file ijms-26-09403-s001.zip › Supplementary Figure S2.jpg]

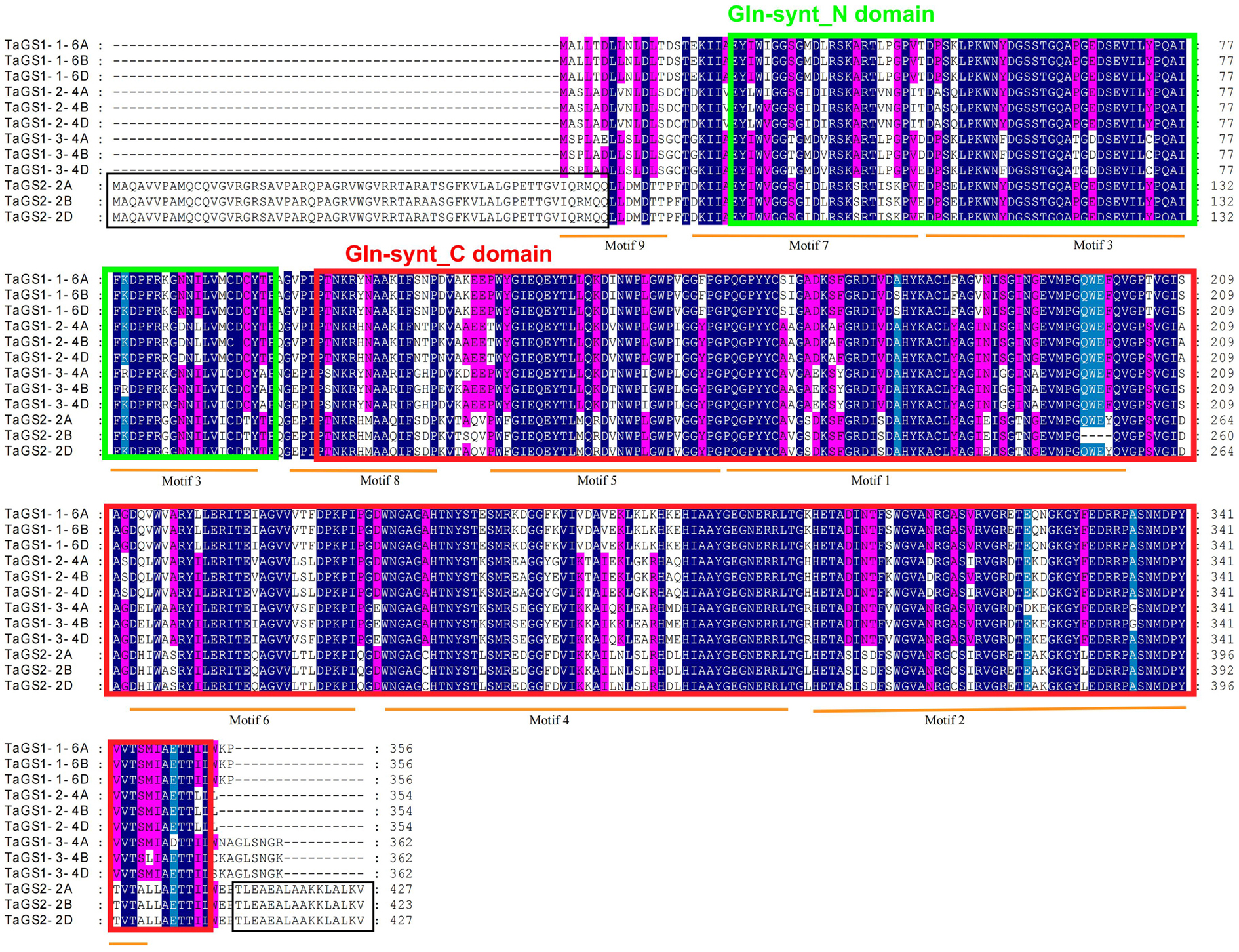

Supplement: Supplementary file 1 [file ijms-26-09403-s001.zip › Supplementary Figure S3.jpg]

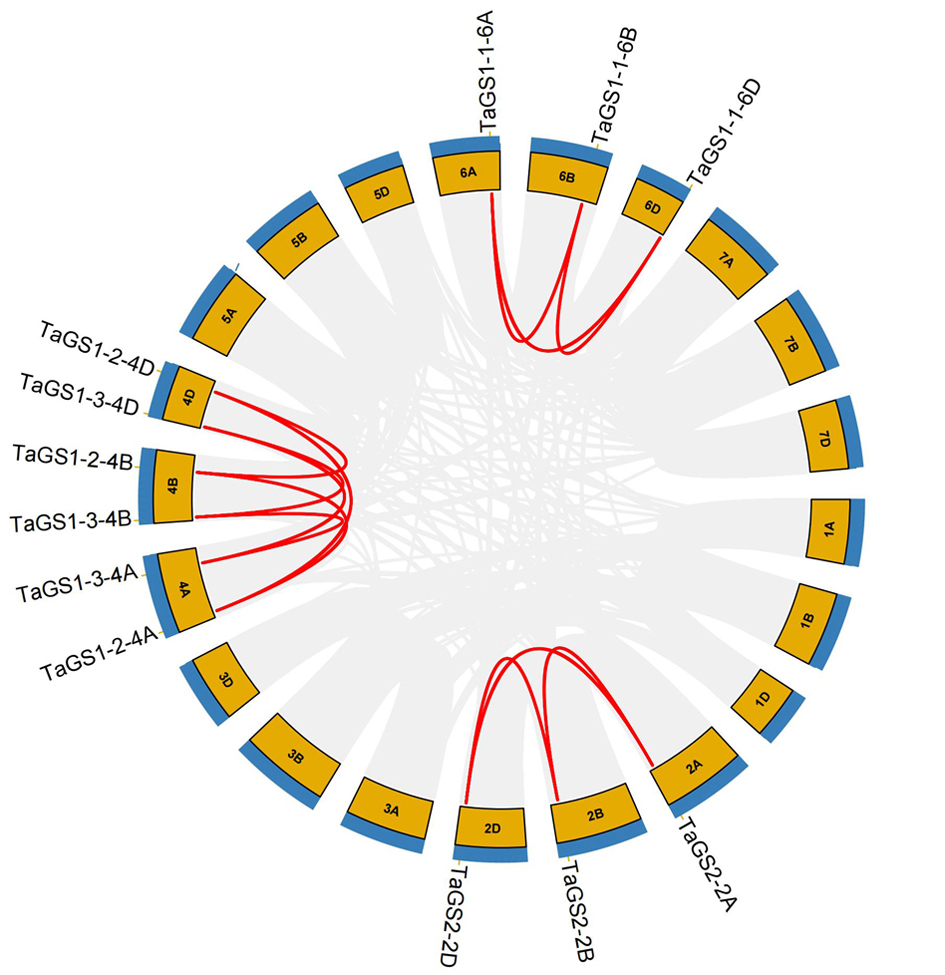

Supplement: Supplementary file 1 [file ijms-26-09403-s001.zip › Supplementary Figure S4.jpg]

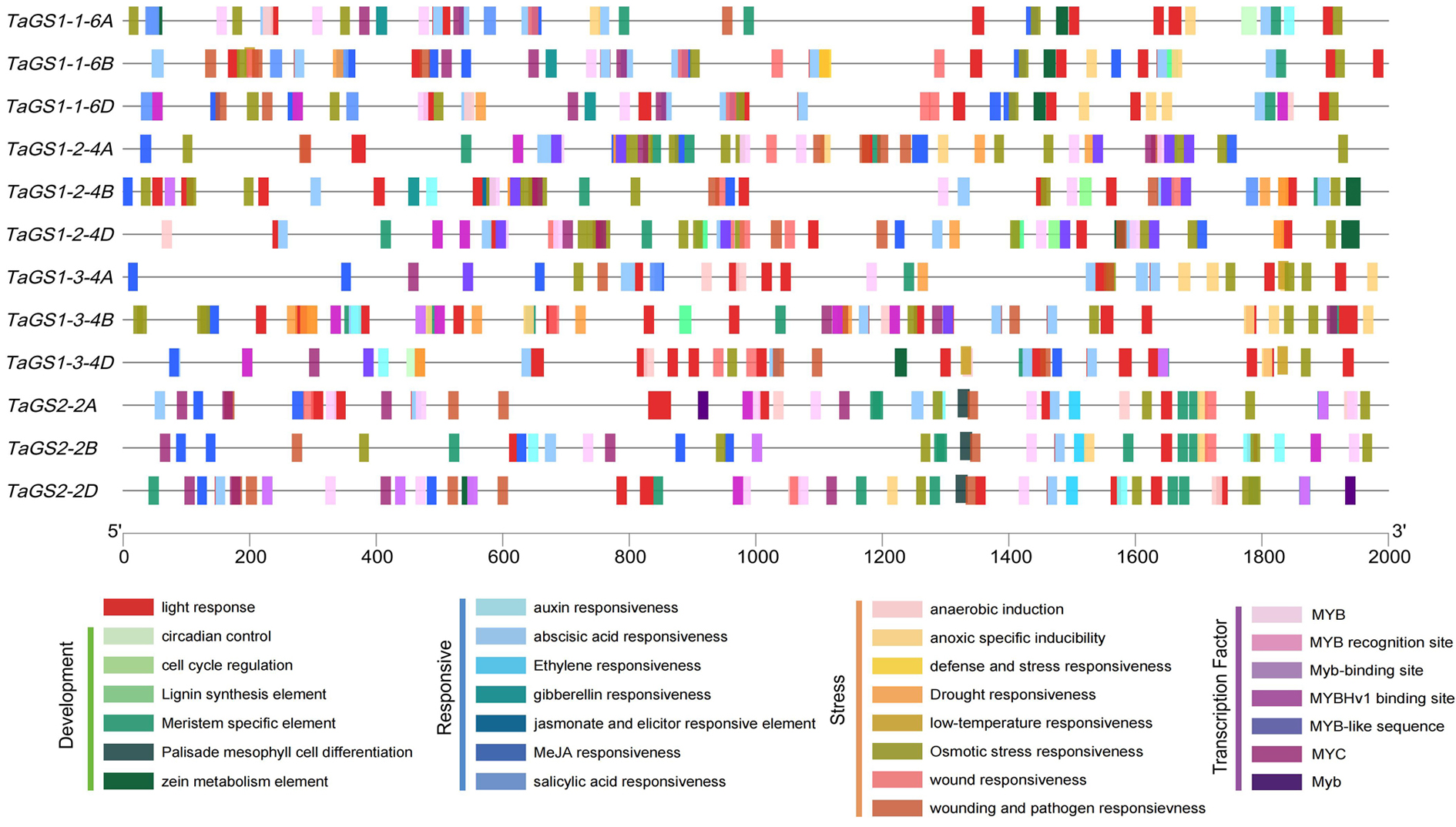

Supplement: Supplementary file 1 [file ijms-26-09403-s001.zip › Supplementary Figure S5.jpg]

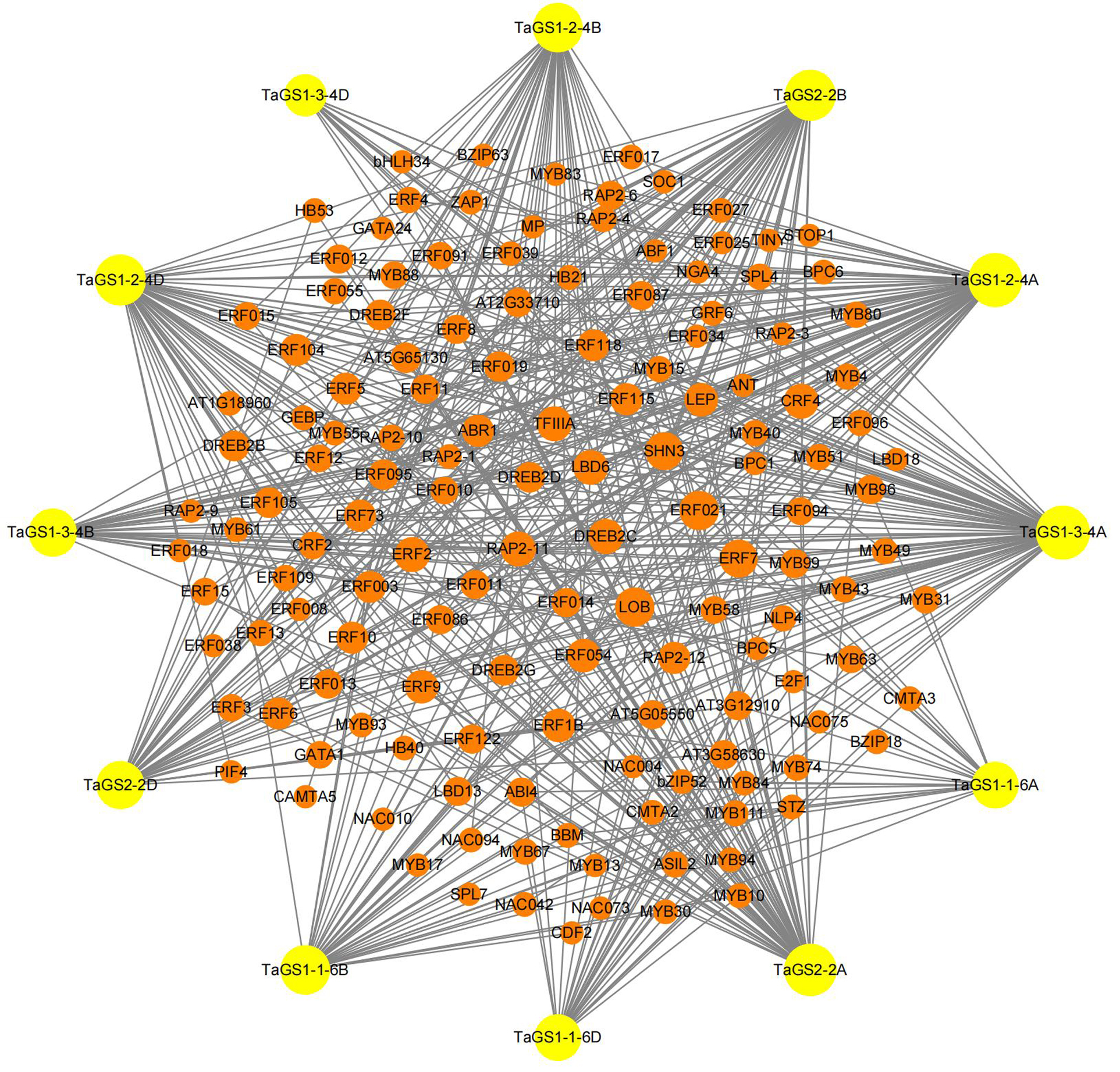

Supplement: Supplementary file 1 [file ijms-26-09403-s001.zip › Supplementary Figure S6.jpg]

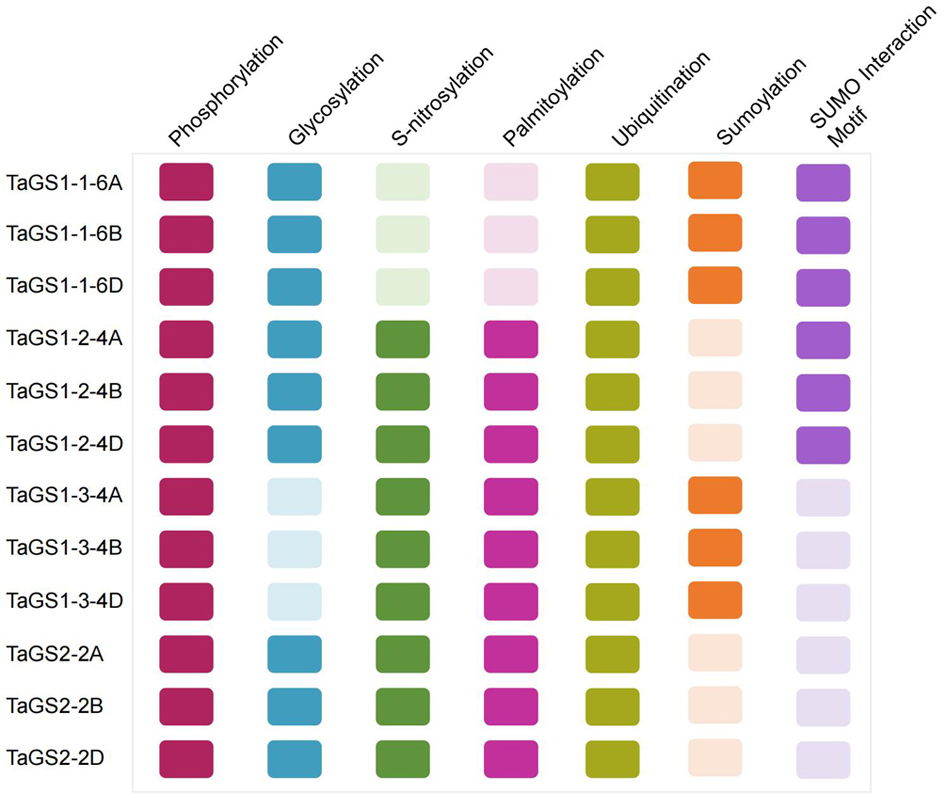

Supplement: Supplementary file 1 [file ijms-26-09403-s001.zip › Supplementary Figure S7.jpg]

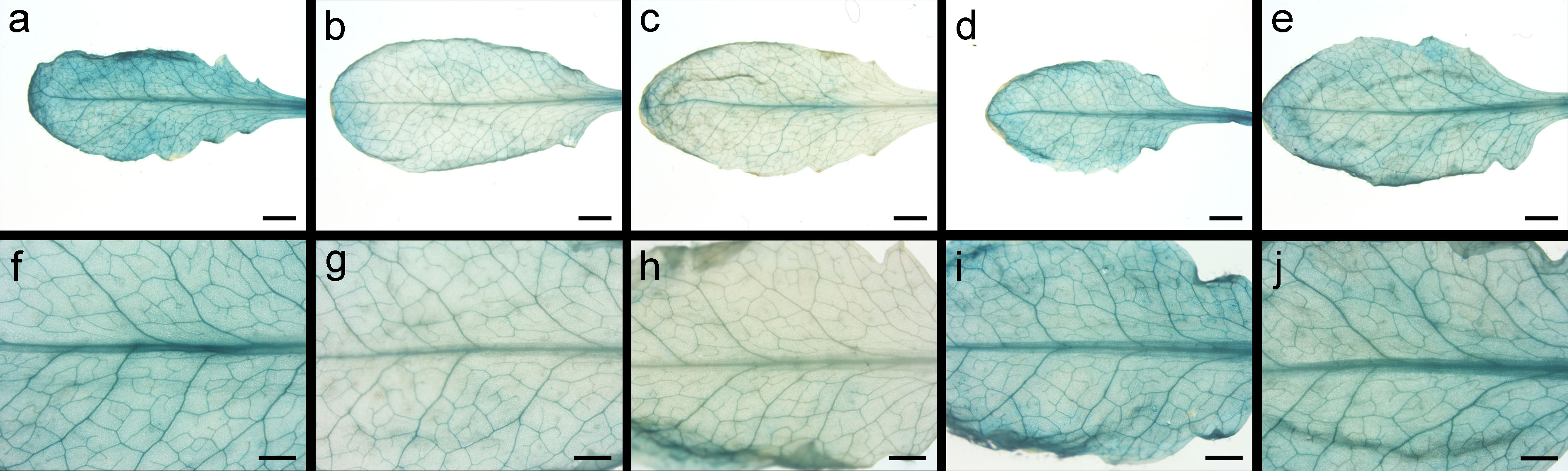

Supplement: Supplementary file 1 [file ijms-26-09403-s001.zip › Supplementary Figure S8.jpg]

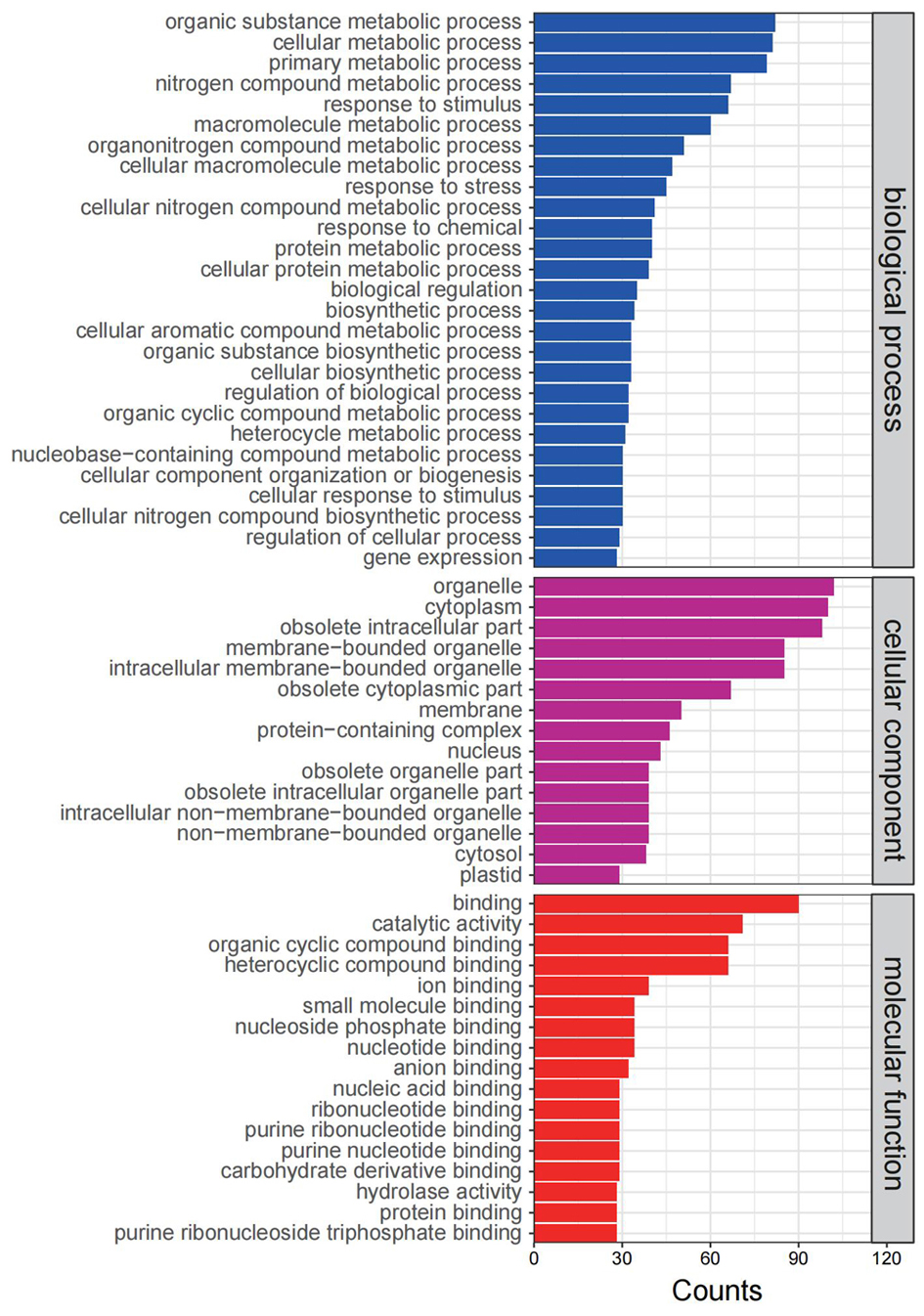

Supplement: Supplementary file 1 [file ijms-26-09403-s001.zip › Supplementary Figure S9.jpg]
